# Supplementary material for: The Anti-Fungal Activity of Nitropropenyl Benzodioxole (NPBD), a Redox-Thiol Oxidant and Tyrosine Phosphatase Inhibitor
Source: Antibiotics (Basel). 2022 Sep 2;11(9):1188. doi: 10.3390/antibiotics11091188 (PMC9495065; doi:10.3390/antibiotics11091188)
Supplement: Supplementary file 1 [file antibiotics-11-01188-s001.zip › antibiotics-1878021-supplementary.pdf]

# The Anti-Fungal Activity of Nitropropenyl Benzodioxole (NPBD), a Redox-Thiol Oxidant and Tyrosine Phosphatase Inhibitor

Gina Nicoletti [1], Kylie White [1],*

[1] STEM College, RMIT University, Melbourne, Victoria, Australia

* Correspondence: kylie.white3@rmit.edu.au

## Supplementary Information

## Supplementary Information S1

**Inhibition of enzymic activity of tyrosine phosphatases**

**Table S1 NPBD and NPFB inhibition of the enzymic activity of human PTP1B and CD45 and bacterial Yop.**

| | PTP1B $IC_{50}$ | CD45 $IC_{15}$ | Yop $IC_{50}$ |
|---|---|---|---|
| NPBD | 3 | 3.7 | 27 |
| NPFB | 57 | >650 | 261 |
| Sodium vanadate | 0.02 | 0.06 | 0.18 |

NPBD an NPFB were tested at 3-fold dilutions 3.7 to 900 µM and control inhibitor sodium vanadate in 10-fold dilutions 10 nM to 100 µM, against 2 mU PTP1B (human), 5 U CD45 (human) and 3 U Yop (bacterial) (Calbiochem) using the ProFluor™ Tyrosine Phosphatase Assay, V1281 (Promega).

Nitropropenyl benzodioxole (NPBD) and nitropropenyl fluorobenzene (NPFB) show selective and dose-dependent inhibition of P TP1B and Yop PTP enzyme function. NPBD is ~9-fold more active than NPFB against PTP1B and ~4.5-fold more active against YOP. NPBD showed low inhibition of human DSP, CD45 [1]. Both compounds show broad antibacterial and antifungal activity [2].

**Supplementary Information S2**

**Table S2 Physico-chemical characteristics of NPBD (nitropropenyl benzodioxole).**

| Parameter | Value |
|---|---|
| IUPAC nomenclature | 5-(2-nitroprop-1-enyl)-1,3-benzodioxole |
| Molecular formula | $C_{10}H_9NO_4$ |
| Optically active | No |
| Stereoisomers | 2 |
| Molecular weight | 207.2 g/mol |
| Appearance | yellow crystalline solid |
| Melting point | 96-98ºC |
| $KD_{(O/W)}$ | 345 |
| Solubility (DMSO) | 300 mg/mL (neutral pH) |
| Solubility (1:1 DMSO:Ethanol) | 150 mg/mL |
| Solubility (1:1 DMSO: Cremophor EL) | 150 mg/mL (with warming) |
| Solubility (100% Ethanol) | 4 mg/mL |
| Solubility (deionized water) | <12 µg/mL |
| Heat resistance | 200°C |
| UV resistance | 4-fold loss in activity by MIC at 4 weeks exposure |
| Stability in solution (DW, DMSO) (dark, 4°C, −20°C) | 12 weeks as measured by control MIC assays |

*Physicochemical studies performed in the Laboratory of Professor Hugh Cornell, RMIT University*

# Supplementary Information S3

## Antifungal activity of NPBD

WE MAKE LIVES BETTER
UT HEALTH SCIENCE CENTER
SAN ANTONIO

F-1692

# Antifungal and Antipneumocystis Activity of the Investigational Antimicrobial BDM-I

A.W. Fothergill[1], M.T. Cushion[2], M.S. Collins[2], W.R. Kirkpatrick[1,3], L.K. Najvar[1,3], T. F. Patterson[1,3], N.P. Wiederhold[1]
The University of Texas Health Science Center at San Antonio[1], Cincinnati Foundation for Biomedical Research and Education[2]
South Texas Veterans Health Care System[3]

S • A • C • M • M
San Antonio Center for Medical Mycology

**Contact Information:** N.P. Wiederhold
UTHSCSA 7703 Floyd Curl Dr., MSC 7750
San Antonio, TX 78229
Tel. (210) 567-4086, e-mail: wiederholdn@uthscsa.edu

### ABSTRACT

**Background:** There is a critical need for the development of new antimicrobials with broad-spectrum activity. In an initial screen, the novel antimicrobial agent BDM-I demonstrated broad-spectrum activity against both fungi and bacteria. Our objective was to further evaluate the in vitro antifungal and antipneumocystis activity of BDM-I against select fungi, including causative agents of opportunistic and endemic mycoses.

**Methods:** Clinical isolates, including *Cryptococcus* species, *Candida glabrata*, *Blastomyces dermatitidis*, *Coccidioides* species, and *Histoplasma capsulatum*, were evaluated. MICs were determined using CLSI reference methods for yeasts (M27-A3) and filamentous fungi (M38-A2). Antipneumocystis activity was measured using a well-described ATP luciferin-luciferase reaction assay, and the IC50 of BDM-I was determined against *P. carinii* and *P. murina*. Mammalian cell toxicity was also measured using the ATP assay in the human lung carcinoma cells (A549) and the rat lung fibroblasts (L2).

**Results:** BDM-I demonstrated potent activity against endemic fungi, including *B. dermatitidis*, *Coccidioides* species, and *H. capsulatum* (MIC90 range 0.25 – 0.5 µg/ml at 100% growth inhibition). Similarly, activity was also observed against *C. neoformans* and *C. gattii* (MIC90 2 µg/ml at 100% growth inhibition) as well as *C. glabrata* (MIC90 2 µg/ml). BDM-I also had marked activity against *P. carinii* and *P. murina* (IC50 on day 3 of exposure <0.1 and 0.174 µg/ml, respectively). While toxicity was observed against the L2 cell line (IC50 <0.1 µg/ml), the antipneumocystis activity was 30 times lower than that observed against the A549 cell line (IC50 0.174 vs. 5.26 µg/ml, respectively).

**Conclusions:** The investigational agent BDM-I has both antifungal and antipneumocystis activity in vitro. Further studies are warranted to determine the potential of this broad-spectrum agent.

| Fungal Species | Cryptococcus neoformans (N=13) | Cryptococcus gattii (N=10) | Candida glabrata (N=10) | Blastomyces dermatitidis (N=10) | Coccidioides species (N=10) | Histoplasma capsulatum (N=10) |
|---|---|---|---|---|---|---|
| 50% Inhibition Endpoint (µg/ml) | | | | | | |
| MIC Range | 0.25 - 2 | 1 - 2 | 0.5 - 1 | 0.06 - 0.125 | 0.125 - 0.25 | ≤0.03 - 0.25 |
| MIC50 | 1 | 2 | 1 | 0.125 | 0.25 | 0.06 |
| MIC90 | 2 | 2 | 1 | 0.125 | 0.25 | 0.25 |
| GM MIC | 1.00 | 1.74 | 0.81 | 0.09 | 0.22 | 0.05 |
| 100% Inhibition Endpoint (µg/ml) | | | | | | |
| MIC Range | 2 | 2 | 1 - 2 | 0.125 - 0.25 | 0.25 | 0.03 - 0.5 |
| MIC50 | 2 | 2 | 1 | 0.25 | 0.25 | 0.125 |
| MIC90 | 2 | 2 | 2 | 0.25 | 0.25 | 0.5 |
| GM MIC | 2.00 | 2.00 | 1.[illegible] | 0.20 | 0.25 | [illegible] |

### BACKGROUND

- The development of novel antimicrobial agents is of critical importance due to evolving antimicrobial resistance.
- BDM-I (**Figure 1**) is a novel antimicrobial agent currently under development by BioDiem Ltd., who has a Non-Clinical Evaluation Agreement with the NIH/NIAID for the pre-clinical evaluation of this investigational agent.
- In initial screens, BDM-I demonstrated broad-spectrum activity against both fungi and bacteria.

### OBJECTIVE

Our objective was to further evaluate the in vitro antifungal and antipneumocystis activity of BDM-I against select fungi. This included causative agents of opportunistic and endemic mycoses and *Pneumocystis* species. In addition, the potential for mammalian cell toxicity was also evaluated.

### MATERIALS AND METHODS

***Isolates***

- Clinical isolates of *Cryptococcus neoformans* and *Cryptococcus gattii*, *Candida glabrata*, *Blastomyces dermatitidis*, *Coccidioides* species, and *Histoplasma capsulatum*, were obtained from the Fungus Testing Laboratory at the UT Health Science Center at San Antonio.
- Isolates of *Pneumocystis carinii* and *Pneumocystis murina* were maintained at the Cincinnati Foundation for Biomedical Research and Education.

***Antifungal Activity***

- In vitro antifungal activity was measured according to the CLSI M27-A3 and M38-A2 guidelines. These assays are performed in the Fungus Testing Laboratory at the UT Health Science Center at San Antonio.
- After the appropriate period of incubation (24 to 48 hours for *Candida glabrata*, 72 hours for *Cryptococcus* species, and 72 – 96 hours for *Blastomyces dermatitidis*, *Coccidioides* species, and *Histoplasma capsulatum*) the MIC values were determined.
- Two MIC values were used: 1) the concentration resulting in a prominent reduction in growth (50% of the growth control), and 2) the concentration resulting in complete inhibition of growth (optically clear well).
- The MICs that inhibited 50% and 90% of the fungi (MIC50 and MIC90, respectively), and the geometric mean (GM) MICs were determined.

***Antipneumocystis Activity***

- Antipneumocystis activity was measured against *P. carinii* and *P. murina*. This work was performed at the Cincinnati Foundation for Biomedical Research and Education.
- For each study, a set of controls was included: 1) growth control (untreated *Pneumocystis*); 2) pentamidine at 0.3 or 1 µg/ml; 3) ampicillin at 10 µg/ml; 4) media control or vehicle control (at the highest concentration used). Plates are incubated at 37°C with 5% $CO_2$ in a water-jacketed incubator.
- At 24, 48, and 72 hours, 50 µl samples were removed for ATP analysis.
- Antipneumocystis activity was measured using an ATP assay (ATPlite luminescence ATP Detection Assay, Perkin Elmer), which is based on the release of bioluminescence driven by ATP in the luciferin-luciferase reaction.
- Activity was classified by the IC50 value as highly active (<0.010 µg/ml), very marked (0.011 - 0.099 µg/ml), marked (0.10 – 0.99 µg/ml), moderate (1.0 – 9.99 µg/ml), slight (10.0 – 49.9 µg/ml), or inactive (≥ 50 µg/ml).

***Mammalian Cell Toxicity***

- The ATP assay described above was used to evaluate the viability of cell monolayers in order to assess for potential toxicity to mammalian cells.
- Confluent monolayers consisted of the human lung cell carcinoma cell line A549 (ATCC CCL-185) and the rat lung fibroblast line L2 (ATCC CCL149).

**Figure 1.** Chemical structure of the novel antimicrobial agent BDM-I.

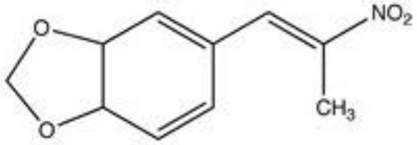


### CONCLUSIONS

The novel antimicrobial agent BDM-I demonstrated in vitro activity against *Cryptococcus neoformans* and *Cryptococcus gattii* as well as the endemic fungi *Blastomyces dermatitidis*, *Coccidioides immitis/posadasii*, and *Histoplasma capsulatum*. This investigational agent also demonstrated marked activity against both *Pneumocystis carinii* and *Pneumocystis murina*. Although toxicity was observed against the rat lung fibroblast line L2, the antipneumocystis activity was 30 times lower than that observed against the human lung cell carcinoma cell line A549. Further studies are warranted to determine the potential of this broad-spectrum antimicrobial agent.

### RESULTS (cont.)

**Table 1.** MIC ranges, MIC50, MIC90 and GM MIC values for BDM-I versus *Cryptococcus* species and endemic fungi.

| Parameter | Cryptococcus neoformans (n = 13) | Cryptococcus gattii (n = 10) | Blastomyces dermatitidis (n = 10) | Histoplasma capsulatum (n = 10) | Coccidioides spp. (n = 10) |
|---|---|---|---|---|---|
| *50% Inhibition Endpoint* | | | | | |
| **MIC Range** | 0.25 - 2 | 1 - 2 | 0.06 - 0.125 | ≤ 0.03 - 0.25 | 0.125 - 0.25 |
| **MIC50** | 1 | 2 | 0.125 | 0.06 | 0.25 |
| **MIC90** | 2 | 2 | 0.125 | 0.25 | 0.25 |
| **GM MIC** | 1.00 | 1.74 | 0.09 | 0.05 | 0.22 |
| *100% Inhibition Endpoint* | | | | | |
| **MIC Range** | 2 | 2 | 0.125 - 0.25 | 0.03 - 0.5 | 0.25 |
| **MIC50** | 2 | 2 | 0.25 | 0.125 | 0.25 |
| **MIC90** | 2 | 2 | 0.25 | 0.5 | 0.25 |
| **GM MIC** | 2.00 | 2.00 | 0.20 | 0.09 | 0.25 |

**Table 2.** Antipneumocystis activity of BDM-I as measured by percent reduction in ATP.

| Species | *Pneumocystis carinii* | | | *Pneumocystis murina* | | |
|---|---|---|---|---|---|---|
| Time Point | Day 1 | Day 2 | Day 3 | Day 1 | Day 2 | Day 3 |
| Media | 0 | 2.04 | 0.84 | 0 | 0 | 12.18 |
| Ampicillin 10µg/ml | 0 | 0 | 0 | 0 | 0 | 12.73 |
| Pent. 1 µg/ml | 75.69 | 96.03 | 83.08 | 78.99 | 78.22 | 71.83 |
| BDM-I 100 µg/ml | 97.30 | 95.74 | 91.92 | 98.85 | 98.76 | 98.77 |
| BDM-I 10 µg/ml | 96.45 | 96.81 | 92.78 | 97.81 | 99.03 | 98.47 |
| BDM-I 1 µg/ml | 53.55 | 90.35 | 90.16 | 63.18 | 98.90 | 97.71 |
| BDM-I 0.1 µg/ml | 34.76 | 10.27 | 73.08 | 26.39 | 21.08 | 21.68 |
| BDM-I IC50 (µg/ml) | 0.047 | 0.411 | < 0.1 | 0.441 | 0.172 | 0.174 |

**Table 3.** Toxicity to mammalian cells as measured by percent reduction in ATP.

| | A549 Cell Line | | | | L2 Cell Line | | | |
|---|---|---|---|---|---|---|---|---|
| Agent | Day 1 | Day 2 | Day 3 | Day 4 | Day 1 | Day 2 | Day 3 | Day 4 |
| Antimycin A 75 µg/mL | 26.79 | 79.34 | 72.56 | 94.85 | 76.37 | 49.59 | 61.84 | 83.05 |
| BDM-I 100 µg/mL | 99.36 | 99.72 | 99.71 | 99.66 | 99.30 | 99.41 | 99.20 | 99.70 |
| BDM-I 10 µg/mL | 11.60 | 31.28 | 52.95 | 39.47 | 99.42 | 99.26 | 99.62 | 99.66 |
| BDM-I 1 µg/mL | 11.17 | 10.45 | 11.77 | 19.85 | 99.15 | 97.19 | 99.59 | 97.08 |
| BDM-I 0.1 µg/mL | 8.06 | 7.87 | 7.46 | 7.07 | 80.37 | 62.54 | 40.30 | 47.16 |
| IC50 (µg/ml) | 13.70 | 8.45 | 5.26 | 6.09 | < 0.1 | < 0.1 | < 0.1 | < 0.1 |

This project has been funded with Federal funds from the NIH/NIAID/DMID Under Contract No. HHSN272201100018I. BDM-I drug-substance was provided by BioDiem Ltd., Melbourne, Australia.

*NPBD, as BDM-I, is in preclinical development by Opal Sciences, a subsidiary of Biodiem Ltd.*

## Supplementary Information S4

### Inhibition of non-small cell lung cancer tumour cell line

The cytoxicity of nitropropenyl benzodioxole (NPBD) and nitropropenyl fluorobenzene (NPFB, Figure S1) for human non-small cell lung cancer cell line A549 and human neonatal foreskin fibroblasts (FF) was assayed using the Sulforhodamine B assay [3]. Cell viability is assessed by the amount of SRB dye bound to cell protein as a proxy for cell mass. Growth Inhibition ($GI_{50}$) is reported as the drug concentration that results in a 50% reduction in the net cellular protein increase in control cells.

The assays were performed by Dr Carleen Cullinane, Peter McCallum Cancer Centre, Cancer therapeutics Program. Translational Research Laboratory, 305 Grattan St, Melbourne, Vic.

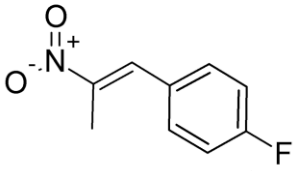


**Figure S1 Molecular structure of NPFB (1-fluoro-4-(2-nitroprop-1-enyl]benzene)**

NPBD showed differential toxicity to human NSCLC A549 cells (mean $GI_{50}$ 1.7 μM) compared to neonatal foreskin fibroblasts ($GI_{50}$ 12.5 μM (Table S3). NPFB was more toxic than NPBD to FF and showed no selective toxicity to the lung cancer cell line.

**Table S3 NPBD inhibition of NSCLC cell line A549 using SRB cytotoxicity assay**

| Compound | Cell line | $GI_{50}$ (μM)[1] |
|---|---|---|
| NPBD | A549[2] | 1.4, 2.1 |
| | FF[3] | 10, 15 |
| NPFB | A549 | 2.2, 1.8 |
| | FF | 2.1, 1.7, 1.6, 2.1 |

The percentage of cell growth is calculated at each drug concentration as: [(Ti-Tz)/(C-Tz)] x 100 for concentrations where Ti ≥ Tz  or  [(Ti-Tz)/Tz] × 100 for concentrations where Ti < Tz.   Tz = absorbance for time zero growth, Ti is test drug growth and C is growth of FF.  $GI_{50}$ is the concentration required to achieve at 72 h a 50% reduction in the net cellular protein increase in control cells.

## Supplementary Information S5

Studies were performed at CDCO Centre for Drug Candidate Optimisation, Monash Institute of Pharmaceutical Sciences, Monash University. 381 Royal Parade, Parkville,VIC 3052, Australia June 2011

### Summary of report and additional author comments

#### Direct inhibition (DI) of CYP isoforms in human liver microsomes

The effect of NPBD on CYP isoforms was measured by assay of substrate-specific interactions in microsomes using known metabolites of each CYP isoform: CYP1A2, CYP2C9, CYP2C19, CYP2D6 and CYP3A4/5) NPBD) showed inhibition of CYP1A2 at $IC_{50}$ 0.53 μM) and 35%% inhibition of CYP2C9 and no inhibition at 20 μM of CYP2C19, -2D6 and -3A4/5 .

**Table S4.** IC50 values against CYP isoforms using the substrate specific approach in human liver microsomes.

| Compound | $IC_{50}$ (μM) [a] | | | | | |
|---|---|---|---|---|---|---|
| | CYP1A2 | CYP2C9 | CYP2C19 | CYP2D6 | CYP3A4/5 (Midazolam 1'-hydroxylation) | CYP3A4/5 (Testosterone 6β-hydroxylation) |
| Reference inhibitor | 2.28 furafylline | 0.33 sulfaphenazole | 1.45 ticlopidine | 0.023 quinidine | 0.021 ketoconazole | 0.023 ketoconazole |
| NPBD | 0.53 | >20 (35%) | >20 (n.m.i.) | >20 (n.m.i.) | >20 (n.m.i.) | >20 (n.m.i.) |

Concentrations of NPBD and reference inhibitors) were incubated (37°C) for 4–40 min with isoform-specific substrates in human liver microsomes. Reaction was initiated by NADPH-regenerating system and quenched by acetonitrile + 0.15 μg/mL of diazepam internal standard. Concentrations of the metabolite specific for each CYP were determined by UPLC-MS. The inhibition of test compound/reference inhibitor was assessed as %reduction in the specific CYP450-mediated metabolite at each concentration relative to the metabolite formation without inhibitor. The $IC_{50}$ is the concentration giving a 50% reduction in the amount of metabolite relative to the maximal formation. Where < 50% inhibition was observed at 20 μM (highest concentration tested) the $IC_{50}$ value is deemed to be >20 μM and the % inhibition indicated in parentheses. n.m.i. No measurable inhibition. *CDCO Centre for Drug Candidate Optimisation, Monash Institute of Pharmaceutical Sciences, Monash University.*

#### Time-dependent inhibition (TPI) of CYP isoforms in human liver microsomes

Time dependent inhibition can occur as a result of irreversible binding to a CYP450 enzyme (mechanism-based) or by CYP inhibition by a metabolite of the compound. Metabolites of BDM-I were not detected in liver

microsomes (data not presented here). The $IC_{50}$ values for all CYP isoforms following pre-incubation with and without NADPH were similar suggesting that NPBD does not act as a mechanism-based inhibitor (Table 2). The $IC_{50}$ values against CYPs 2C9, 2C19 and CYP3A4/5 in the presence of BDM-I were lower in the TDI assay than the DI assay suggesting 30 min pre-incubation with and without NADPH resulted in increased inhibition of CYPs. This increase in inhibition was independent of the presence or absence of NADPH suggestive of a non-CYP-mediated process. BDMH exhibited non-CYP-mediated degradation in liver microsomes (data not presented). The increase in CYP inhibition may be due to products of cofactor-independent degradation of NPBD or direct reactivity of NPBD during the pre-incubation period, such as with thiol-containing biomolecules.

**Table S5.** $IC_{50}$ values for NPBD and reference inhibitors against CYP isoforms after 30 min pre-incubation in the absence and presence of NADPH in human liver microsomes.

| CYP Isoform (Metabolic pathway) | Compound | $IC_{50}$ (μM) [a] Pre-incubation Condition | | Fold shift in $IC_{50}$ [b] |
|---|---|---|---|---|
| | | - NADPH | + NADPH | |
| **CYP1A2** (phenacetin-O-deethylation) | **NPBD** | 0.91 | 0.93 | 1.0 |
| | **Furafylline** (Ref. MB inhibitor) | 5.63 | 0.29 | 19.5 |
| | **Ketoconazole** (Ref. direct inhibitor) | >45 (41%) | >45 (48%) | c.n.c. |
| **CYP2C9** (tolbutamide methylhydroxylation) | **NPBD** | 19.8 | >20 (45%) | <1.0 |
| | **Tienilic acid** (Ref. MB inhibitor) | 1.46 | 0.21 | 6.9 |
| | **Sulfaphenazole** (Ref. direct inhibitor) | 0.56 | 0.62 | 0.9 |
| **CYP2C19** ((*S*)-mephenytoin-4'hydroxylation) | **NPBD** | 16.7 | 19.3 | 0.9 |
| | **Ticlopidine** (Ref. MB inhibitor) | 1.32 | 0.42 | 3.2 |
| | **Ketoconazole** (Ref. direct inhibitor) | 7.2 | 5.5 | 1.3 |
| **CYP2D6** (dextromethorphan-*O*-demethylation) | **NPBD** | >20 (n.m.i.) | >20 (21%) | c.n.c. |
| | **Paroxetine** (Ref. MB inhibitor) | 0.38 | 0.11 | 3.4 |
| | **Quinidine** (Ref. direct inhibitor) | 0.024 | 0.026 | 0.9 |
| **CYP3A4/5** (midazolam-1'-hydroxylation) | **NPBD** | 8.1 | 9.5 | 0.9 |
| | **Verapamil** (Ref. MB inhibitor) | 11.3 | 4.3 | 2.6 |
| | **Ketoconazole** (Ref. direct inhibitor) | 0.018 | 0.023 | 0.8 |
| **CYP3A4/5** (testosterone 6β-hydroxylation) | **NPBD** | 5.9 | 8.4 | 0.7 |
| | **Verapamil** (Ref. MB inhibitor) | 35.0 | 7.9 | 4.4 |
| | **Ketoconazole** (Ref. direct inhibitor) | 0.044 | 0.058 | 0.8 |

a) < 50% inhibition reported as $IC_{50}$ >50% for the respective highest concentration with % inhibition in parentheses. b) Calculated as $IC_{50}$ (-NADPH) / $IC_{50}$ (+NADPH). c.n.c. Could not calculate.

BDM-I or reference inhibitors concentrations were pre-incubated at 37°C in human liver microsomes for 30 min at 10-fold the assay target concentration in the absence or presence of NADPH. Aliquots were diluted 10× in buffers (×5) containing the probe substrate for each CYP isoform. NADPH-regenerating system was added to each assay and incubated at 37°C for 10 - 40 min and the interaction quenched by addition of ice-cold acetonitrile with 0.15 μg/mL of diazepam. Samples were centrifuged and concentrations of the CYP-specific metabolite in the supernatant were determined by UPLC-MS relative to calibration standards prepared in microsomal matrix [4,5]. *CDCO Centre for Drug Candidate Optimisation, Monash Institute of Pharmaceutical Sciences, Monash University.*

**Supplementary Information S6**

**Toxicology studies for NPBD (Compound 1) in rodents.**

## A Oral absorption of Compound (1) in rats after a single dose.

*Non-GLP Study conducted at RMIT University Animal Facility Bundoora West Vic Australia (GLP accredited).*

***Test protocol***. Sprague-Dawley rats (6 w/o, 5×) were administered aqueous suspensions (~100 mL/kg by gavage) of Compound (1) in sterile LPW (100, 500, 1000 and 1250 mg/kg). Blood (100-200 μL) was removed from the tail at 4 and 8 hours. Compound (1) was extracted (×2) from serum by toluene and absorbance measured at 370 nm (Hitachi U2000). A spiked control, 100 μg/mL Compound (1) in 50% methanol/water (V/V) and LPW controls were assayed. Animal observations were recorded twice daily for 7 days. Sacrifice and necropsy were performed at 7 days. Gross pathology was recorded and samples of heart, lung, liver, kidney, stomach, spleen, duodenum and colon removed (10% formalin) for histology.

***Observations.*** At doses of 100 and 500 mg/kg there were no adverse symptoms over the 7-day study period and tissues at necropsy looked normal. One rat (1000 mg/kg) was euthanased at 28 h. Postmortem examination revealed spotty and congested lung and bloated stomach. Liver heart and kidney appeared normal but histology was indicative of dilated cardiomyopathy. Remaining 4 rats showed no visible lesions except for some lung congestion. One rat dosed at 1250 mg/kg was distressed and euthanized at 8 h. Lung congestion was noted. Histology showed mild congestion in the liver, lung and myocardium associated with some sinusoidal cell degeneration. Remaining rats had no visible lesions and appeared to tolerate treatment.

Compound (1) was well tolerated at single doses (NOEL <1000 mg/kg bw) and absorption was ~2.5% for all doses

## B Acute and 7 day repeat dose toxicity in mice after oral dosing of Compound (1)

*Non-GLP study conducted at RMIT University Animal Facility Bundoora West, Vic, Australia, GLP accredited animal laboratory.*

**Acute dosing:** Single doses of Compound (1) in warmed Canola oil at 50, 100 and 200 mg/kg and Canola oil control were administered by oral gavage (vol ≤150 μL) to fasted female BalbC mice (6-8 wo, 5 per treatment). Mice were observed for 7 days then sacrificed and necropsied and observed for gross pathology.

**7 day repeat dosing:** BalbC mice (5 per group) were dosed daily in the morning for 7 days with a single dose of either Compound (1) at 10, 25 and 100 mg/kg or canola oil alone. animals were observed daily and at 7 days sacrificed and necropsied for gross pathology.

**Observations.** NPBD was well tolerated on daily repeat dosing to 7 days at 100 mg/kg and no adverse effects were seen on gross pathology at 7 days. NOEL >100 mg/kg/day. Compound (1) in canola oil was well tolerated by mice on single oral dosing at 200 mg/kg and no adverse effects were seen on gross pathology at 7 days post dosing. NOEL 200 mg/kg.

## C 7 day repeat dose-range finding study in rats

*GLP study in accordance with ICH guidelines.*

**Protocol:** Compound (1) was formulated as a suspension in 1% CMC and 0.05% Tween 80. Rats (5 male, 5 female per treatment) received vehicle or Compound (1) by oral gavage (0, 300, 1100 & 2000 mg/kg), once daily for 7 days. Animals were monitored daily for clinical toxicity, morbidity or mortality. Each animal was subject to a gross necropsy, and a standard tissue list was retained in 10% formalin for possible histopathology. Blood samples for evaluation of serum chemistry, haematology and coagulation parameters were collected from all animals prior to terminal sacrifice on Day 8.

**Results summary:**

Dosing at 1150 mg/kg/day showed 6/10 mortality, 4 animals were found dead and 2 were moribund and sacrificed Mortality at 2000 mg/kg/day was 100%, 9/10 animals being moribund and sacrificed and one found dead on Day 6. All rats on 300 mg/kg survived to sacrifice on Day 8.

Clinical signs of toxicity for 1150 and 2000 mg/kg/day included brown staining around anus, soft faeces, red staining of the cage paper, abdomen and nares, decreased activity, and ruffled haircoat. Moribund sacrificed animals showed clinical signs of toxicity, gross pathological changes on necropsy and changes in haematological and clinical chemistry parameters (data not presented).

There were some clinical signs in male rats at 300 mg/kg/day, haematological changes in female rats and no changes in clinical chemistry parameters in any animal. Gross necropsy pathology appeared normal but females had increased organ weights (data not presented). The no-observed adverse-effect level (NOAEL) was <300 mg/kg/day and the lowest lethal dose (LLD) was <1150 mg/kg/day.

## Supplementary Information S7

### Maximum tolerated dose in nude mouse

*Peter McCallum Cancer Centre, Cancer therapeutics Program*

*Translational Research Laboratory, 305 Grattan St, Melbourne, Vic. Australia*

*Dr. Carleen Cullinane*

### Method

NPBD (100 mg/mL in DMSO) formulated stock solutions: Vehicle 1: 2-4 mg/mL; 12.5% DMSO, 12.5% ethanol, 12.5% Chremophor EL, 62.5% saline for 1, 5, 10, 15, 20 mg/kg doses. Vehicle 2: 5 mg/mL; 15%DMSO, 12.5% ethanol, 12.5% Chremophor El for 25 mg/kg dose. Stock solutions were formulated by addition of the components in order immediately prior to use. Groups of two female Balb/c nude mice (Animal Resources Centre, Western Australia) were given BDM-I (0. 1. 5. 10 15, 20 and 25 mg/kg) at a rate of 0.05 ml/10 g body weight) by intravenous injection twice weekly for three weeks. Mice were weighed daily and monitored for signs of toxicity. Animals were given a feed supplement (Ensure) daily. Blood samples (100-200 mL from tail) were taken 2 days after Week 3 last dose. BDM-I was extracted (×2) from serum by toluene and absorbance measured at 370 nm (RMIT).

### Results

Mice were temporarily subdued and wobbly for 5-10 minutes post injections, probably due to osmotic shock. Dosing from 0 to 20 mg/kg using Vehicle 1 when given twice weekly did not adversely affect mouse weight or general health and well-being (Figure S2). The 25 mg/kg dose in Vehicle 2 was also well tolerated (Figure S3). Observed increases in mouse weight is likely to be due to the effects of the feed supplement.

No Maximum Tolerated Dose was established due to the inability to solubilise the compound for IV delivery at concentrations higher than 25 mg/kg mouse weight. Nude mice tolerated 25 mg/kg well

after twice weekly dosing for 3 weeks.

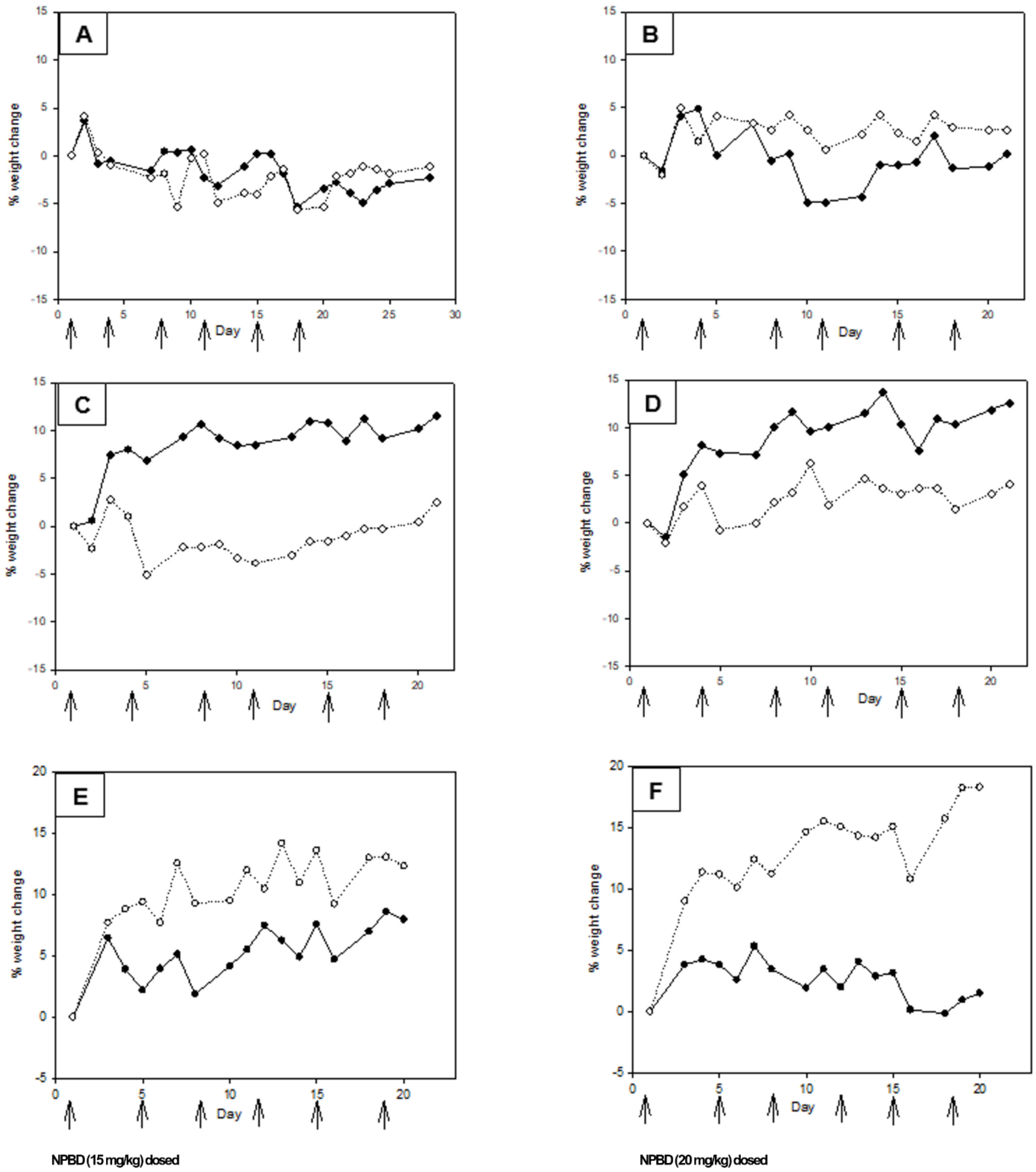


**Figure S2.** Percent weight change associated with twice weekly dosing of NPBD at 0 (A), 1 (B), 5 (C), 10 (D), 15 (E) and 20 (F) mg/kg. Each symbol represents an individual animal. Arrows represent days on which the compound was administered.

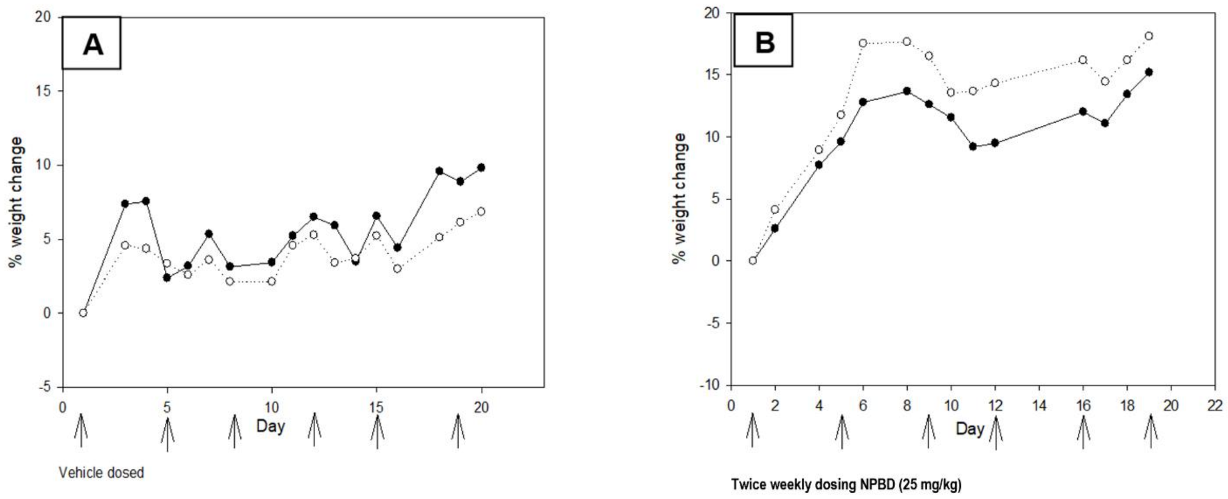


**Figure S3.** Percent weight change associated with twice weekly dosing of 0 (A) and 25 (B) mg/kg NPBD in vehicle containing 15 % DMSO, 12.5 % ethanol, 12.5 % Cremophor and 60 % ethanol. Each symbol represents an individual animal. Arrows represent days on which the compound was administered.

## References

1. White, K.S. The antimicrobial mechanism of action of 3,4-methylenedioxy-β-nitropropene. PhD Thesis, RMIT University, Melbourne, 2008.
2. Nicoletti, G.; Cornell, H.J.; Hugel, H.M.; White, K.S.; Nguyen, T.; Zalizniak, L.; Nugegoda, D. Synthesis and antimicrobial activity of nitroalkenyl arenes. *Anti-Infective Agents* **2013**, *11*, 179-191.
3. Skehan, P.; Storeng, R.; Scudiero, D.; Monks, A.; McMahon, J.; Vistica, D.; Warren, J.T.; Bokesch, H.; Kenney, S.; Boyd, M.R. New Colorimetric Cytotoxicity Assay for Anticancer-Drug Screening. *JNCI: Journal of the National Cancer Institute* **1990**, *82*, 1107-1112, doi:10.1093/jnci/82.13.1107.
4. Obach, R.S.; Walsky, R.L.; Venkatakrishnan, K. Mechanism-Based Inactivation of Human Cytochrome P450 Enzymes and the Prediction of Drug-Drug Interactions. *Drug Metabolism and Disposition* **2007**, *35*, 246-255, doi:10.1124/dmd.106.012633.
5. Walsky, R.L.; Obach, R.S. Validated assays for human cytochrome P450 activities. *Drug Metabolism and Disposition* **2004**, *32*, 647-660, doi:10.1124/dmd.32.6.647.
